# Evolution and Expression Plasticity of Opsin Genes in a Fig Pollinator, *Ceratosolen solmsi*

**DOI:** 10.1371/journal.pone.0053907

**Published:** 2013-01-16

**Authors:** Bo Wang, Jin-Hua Xiao, Sheng-Nan Bian, Li-Ming Niu, Robert W. Murphy, Da-Wei Huang

**Affiliations:** 1 Key Laboratory of Zoological Systematics and Evolution, Institute of Zoology, Chinese Academy of Sciences, Beijing, China; 2 Graduate School of the Chinese Academy of Sciences, Beijing, China; 3 Plant Protection College, Shandong Agricultural University, Tai’an, China; 4 Environment and Plant Protection Institute, Chinese Academy of Tropical Agricultural Sciences, Danzhou, Hainan, China; 5 State Key Laboratory of Genetic Resources and Evolution, Kunming Institute of Zoology, Chinese Academy of Sciences, Kunming, China; 6 Department of Natural History, Royal Ontario Museum, Toronto, Ontario, Canada; George Washington University, United States of America

## Abstract

Figs and fig pollinators have co-evolved species-specific systems of mutualism. So far, it was unknown how visual opsin genes of pollinators have evolved in the light conditions inside their host figs. We cloned intact full-length mRNA sequences of four opsin genes from a species of fig pollinator, *Ceratosolen solmsi*, and tested for selective pressure and expressional plasticity of these genes. Molecular evolutionary analysis indicated that the four opsin genes evolved under different selective constraints. Subsets of codons in the two long wavelength sensitive opsin (LW1, LW2) genes were positively selected in ancestral fig pollinators. The ultraviolet sensitive opsin (UV) gene was under strong purifying selection, whereas a relaxation of selective constrains occurred on several amino acids in the blue opsin. RT-qPCR analysis suggested that female and male fig pollinators had different expression patterns possibly due to their distinct lifestyles and different responses to light within the syconia. Co-evolutionary history with figs might have influenced the evolution and expression plasticity of opsin genes in fig pollinators.

## Introduction

Opsin genes encode proteins that are members of the G protein coupled receptors (GPCR). These proteins, which have molecular masses of 30–50 kDa, can form visual pigments with a covalently bonded light-absorbing chromophore, typically the 11-*cis*-retinal chromophore [1]–[8]. In animals, photosensitivity, which serves many purposes including vision, is mainly conferred by visual pigments [9]. Insects occur in various habitats and experience a great diversity of visual conditions. Not surprising, insects have a concomitantly diverse array of visual receptors [2]. Many studies reveal adaptations of insects to their specific light environment or behavior by analyzing the opsin genes [10]–[20].

Figs (family Moraceae) and their insect pollinators (family Agaonidae) form one of the best known examples of obligate mutualism [21]. Female wasps enter receptive fruits (syconium), the unique, closed inflorescence of figs, to lay eggs and the process results in the pollination of flowers [22]. As the larvae develop, they induce galls. Male fig wasps cannot leave the cavity of fig fruits, whereas the new generation of female pollinators is responsible for colonizing new hosts [22], [23]. This fine-tuned mutualistic system is at least tens of millions of years old [24]–[26]. Sex-specific morphological and behavioral specializations in the fig wasps are associated with life cycles of their hosts [22], [23]. The most remarkable specialization is the extreme extent of sexual dimorphism. Female pollinators possess functional wings and compound eyes, while males are apterous and have vestigial compound eyes and antennae. Additionally, ocelli are absent in male pollinators [23].

Phenotypic adaptation has a genetic basis and this should be reflected in gene sequences and/or patterns of gene expression. Herein, we explore how opsin genes of fig pollinators have evolved and are expressed. Molecular evolutionary analyses and reverse transcription quantitative PCR (RT-qPCR) experiments on *Ceratosolen solmsi* are employed to answer a suite of questions in fig pollinators. (1) What opsin genes do fig pollinators express? (2) How did opsin gene sequences evolve in fig pollinators? (3) What are the circadian rhythms of opsin gene expression in fig pollinators? (4) Does the expression of opsin genes differ between females and males and if so how?

## Materials and Methods

### Ethics Statement

The sampling of living material involved in our experiments includes figs (*Ficus hispida*) and fig pollinators (*C. solmsi*). All necessary permits were obtained for the field sampling. Collection permits were provided by the Institute of Environment and Plant Protection, Chinese Academy of Tropical Agricultural Sciences.

### Sample Collection and Experimental Design

Fig fruits of *F. hispida* were collected from Danzhou (N.19°30′29′′, E.109°29′6′′), Hainan province, China in October 2010 and October 2011. All fig fruits were collected at the same developmental stage several days before becoming ripe. Identity of the fig pollinator *C. solmsi* was confirmed using morphological traits ascertained with a Nikon SMZ80 microscope.

We subjected the fig fruits to different treatments of light and then collected adult fig pollinators. Half of the fig fruits were kept in a darkroom (0:24 L:D), and the others were kept in an environmental chamber for light treatment with a daily light cycle (∼15:9 L:D). After two days of treatments, every 3 hours (3:00, 6:00, 9:00, 12:00, 15:00, 18:00, 21:00, 24:00) figs were flash-frozen in liquid nitrogen. Subsequently, female and male pollinators were removed from the inside of the syconia and immersed into Sample Protector (TaKaRa, China). In addition, to evaluate how opsin gene expression changes outside the fig fruits, we also collected females that had emerged from the syconia under light treatment at each time point and exposed them to light for 3 additional hours before flash-freezing. Males were not submitted to this treatment because they seldom emerge from the syconia. Individuals for all insect samples were at the same developmental stage. In total, 40 groups (3 female and 2 male samples for each of the 8 different sampling time points) were collected as follows: emerged females exposed to light outside figs for 3 h (emerged females light); females (fig-females light) and males (fig-males light) from inside of light treated fig fruits(∼15:9 L:D); females (fig-females dark) and males (fig-males dark) from inside of dark treated fig fruits.

### RNA Isolation and cDNA Synthesis

For each of the 40 sampling groups, total RNA was extracted using the EasyPure™ RNA kit (TransGen Biotech, Beijing, China) and dissolved in RNase-free water. Because fig pollinators are very small, we used 40 whole-body individuals for each RNA sample. Genomic DNA was removed by treating with DNase I according to the manufacturer’s instruction. A NanoDrop-2000 Spectrophotometer (Thermo, Madison, WI, USA) was used to measure RNA purity (A_260_/A_280_) and concentration. In total, 120 RNA samples (3 biological replicates for each group) with values of A_260_/A_280_ between 1.9 and 2.2 and an A_260_/A_230_ ratio of more than 2.0 were selected for further experiments. The integrity of the RNA samples was evaluated by electrophoresis on 1.0% agarose gels stained with ethidium bromide. Single-stranded cDNA was synthesized from 1 µg total RNA with oligo-dT per 20 µl reaction using TransScript II First-Strand cDNA Synthesis SuperMix (TransGen Biotech, Beijing, China).

### RT-PCR, RACE-PCR, and Sequencing

Nested RT-PCR and semi-nested RT-PCR were performed to amplify the partial coding sequences of the opsin genes. For each PCR reaction, a volume of 25 µl contained 0.2 mM of each dNTP, 0.2 µM of each primer, and 5 activity units of Transtaq™ DNA Polymerase High Fidelity (TransGen Biotech, Beijing, China). The first round of PCR was performed starting with 5 min at 95°C followed by 35 cycles of 30 s at 95°C, 45 s at 50°C, and 1 min at 72°C; and a final step of 10 min at 72°C. The second round PCR was conducted using the same program with a 0.2% final concentration of the first round PCR product. Subsequently 3′ and 5′ RACEs were conducted to obtain the full length mRNA sequences using the SMART RACE cDNA Amplification Kit (Clontech, Mountain View, CA, USA). Different gene-specific primers were used in combination with the universal primer (UPM). The PCR conditions comprised 3 min at 95°C followed by 5 cycles of 5 s at 95°C, 2 min at 72°C; 5cycles of 5 s at 95°C, 30s at 70°C, and 1.5 min at 72°C and then 30 cycles of 5 s at 95°C, 30 s at 68°C, and 1.5 min at 72°C; 10 min at 72°C. A second round semi-nested RACE-PCR was performed when no specific band was observed by electrophoresis in the first round. All PCR reactions were carried out on an Applied Biosystems Veriti™ 96-Well Thermal Cycler (ABI, Foster City, CA USA). The primers used for RT-PCR and RACE-PCR (Table S1) were designed using Primer Premier 5.0 [27]. Purified PCR products were cloned with the pEASY-T3 cloning kit (TransGen Biotech, Beijing, China) and three positive clones were sequenced with primer M13 by Biosune Sequencing Center, Beijing, China.

### Phylogenetic Inference and Molecular Evolutionary Analysis

Full-length coding sequences of opsins from *C. solmsi* were aligned with opsins from several representative insects (Table S2) using ClustalW implemented in MEGA 5.0 [28]. A maximum likelihood (ML) tree was constructed by PhyML 3.0 [29] with the best fit model of nucleotide evolution determined by jModeltest [30]. One hundred ML bootsrap replicates were obtained to assess clade robustness. We used two opsins from cephalopods, *Octopus dofleini* and *Sepia officinalis*, as the outgroup, according to a previous study that showed opsins from mollusks were suitable to root arthropod opsins [8]. In addition, we constructed ML trees for all four opsin genes from hymenopteran insects using corresponding sequences from a hemimetabolic insect, *Dianemobius nigrofasciatus*, as the outgroup.

We used the Codeml program implemented in PAML 4.5 [31] to test for selection pressure on the background branch of hymenopterans (Table S2). Potential positive selection was tested based on the ratio (ω) of nonsynonymous (K_a_) to synonymous (K_s_) substitutions rates (ω = K_a_/K_s_). Generally, ω<1 was assumed to indicate purifying selection, ω = 1 indicated neutral evolution, and ω >1 indicated positive selection. For each gene, several tests were conducted using different models. M0 (one-ratio model) assumed an invariant ω value and measured natural selection acting on a specified protein. Site-specific models were as follows: M1a (nearly neutral), M2a (positive selection), M7 (beta), M8 (beta&ω), and M8a (beta&ωs = 1), which allowed the ω ratio to vary among codons, were used for site-by-site detection of positive selection. Branch model (two-ratio), which allowed ω to vary among branches, was used to detect positive selection acting on particular lineages. Branch-site model, which allowed ω to vary both among sites in the protein and across branches, was used to detect episodic positive selection [32], [33]. For the Branch and Branch-site models, the branch leading to *Ceratosolen solmsi* and the species of *Nasonia* was labeled as the foreground lineage to test whether or not positive selection affected opsins starting from the common ancestor of parasitic wasps. The pairwise comparisons of M1a vs. M2a, M7 vs. M8, Branch model (two-ratio) vs. Branch null model (fixed ω = 1 and ω = 1), and Branch-site model (model = 2, NSsites = 2) vs. Branch-site null model (fixed ω = 1and ω = 1) were used to perform likelihood ratio tests (LRTs) and their significance was assessed using a χ2 distribution. When the LRT was significant, a Bayes Empirical Bayes (BEB) analysis was used to identify positively selected sites.

In addition, we obtained the secondary structure of the LW1 and LW2 opsins by submitting the putative protein sequences to TMHMM Server v2.0 http://www.cbs.dtu.dk/services/TMHMM/ [34]. The positively selected sites were mapped on these reconstructions.

### RT-qPCR Expression Analysis

Previously, we amplified 9 housekeeping genes from *C. solmsi* that were frequently used as reference genes for qPCR studies in insects and systematically evaluated their expression stability(submitted). *RPL13a* (the gene encoding ribosomal protein L13a) and *UBC* (the gene encoding ubiquitin-conjugating enzyme) were selected as the best reference genes for normalizing the RT-qPCR data. Based on the full coding sequences of the opsin genes, four gene-specific primer pairs were designed using Primer Premier 5.0 [27]. The intron positions of each opsin gene are conservative across closely related species, thus we aligned the four genes to genomic opsin genes from *Apis mellifera* and *Nasonia vitripennis* to identify the splicing sites. All primer pairs were designed to span an intron. Amplification efficiencies and R^2^ coefficients of the primer pairs were determined with the slopes of the standard curves generated from plasmid standards. Products obtained via gene-specific primers were cloned with the PEASY-T3 cloning kit (TransGen Biotech, Beijing, China). Clones with appropriate insert size were verified by PCR and sequencing. Plasmids were prepared with EasyPure Plasmid MiniPrep Kit (TransGen Biotech, Beijing, China). We determined the amount of plasmids using a NanoDrop-2000 Spectrophotometer (Thermo, Madison, WI, USA) and calculated the copy numbers of the plasmids. Ten-fold serial dilutions to 10^7^, 10^6^, 10^5^, 10^4^, and 10^3^ copies per 20 µl RT-qPCR reaction were made for each plasmid with two technical replicates to generate standard curves. The formula *E* = 10^ (−1/slope)^ was used to calculate amplification efficiencies (*E*), which reflected the efficacy of each primer pair.

The Stratagene Mx3000p qPCR system (Stratagene, La Jolla, CA) was used to carry out RT-qPCR. A 20 µl PCR mixture containing 1 µl of template, 10 µl TransStart Green qPCR SuperMix UDG(2×) (TransGen Biotech, Beijing, China), 0.4 µl Passive Reference Dye II (50×) (TransGen Biotech, Beijing, China), 0.8 µl primer mix (0.2 µM), and 7.8 µl sterile water was prepared. A template for no-RT control was prepared for each sample. All cDNA templates were stored at −20°C. No-RT controls for all 120 samples were performed to check for gDNA contamination, and a no-template control was also conducted for each run and each gene to preclude reagent contamination. Melting curves were constructed for all runs to confirm amplification specificity. RT-qPCR reactions of all genes for each sample were duplicated (technical replicates) to account for variation between runs. The same thermal conditions were used for all RT-qPCR reactions: 95°C for 10 s, 54°C for 15 s and 72°C for 10 s for 40 cycles.

Several studies indicated that the mean of individual PCR efficiency (*E_m_*) gave more reliable results than a standard curve-derived efficiency [35]–[37]. Thus, we determined the baseline and calculated an *E_m_* of individual reactions for each primer pair from the raw RT-qPCR data using LinRegPCR [38], [39]. Subsequently, the quantification cycle (*C_q_*) and *E_m_* values were used to calculate the relative expression of the four opsin genes with respect to reference genes *RPL13a* and *UBC* according to the following equation:
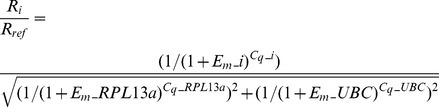



*R_i_* was the expression of each individual opsin gene *i*; *R_ref_* was the expression of the reference genes; *R_i_*/*R_ref_* was the expression of each individual opsin *i* normalized to the reference genes *RPL13a* and *UBC*; *C_q_*_*i* was the quantification cycle value for each individual opsin gene *i*; C*_q_*_*RPL13a* was the quantification cycle value for *RPL13a*; C*_q__UBC* was the quantification cycle value for *UBC*. Additionally, the expression of each opsin gene (*R_i_*/*R_all_*) relative to the total opsin pool, which was more likely to reflect differences in visual sensitivity [40], was also determined as:
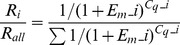
where *R_all_* was the total expression of the four opsin genes.

### Statistical Analysis

A one-way ANOVA was carried out to test for variation in levels of mRNA among time points. Two-way ANOVAs were conducted to evaluate the effect of light and time on opsin gene expression. Cosinor analysis was performed using Cosinor.exe v. 2.3 (Roberto Refinetti, University of South Carolina, Salkehatchie, SC). The significance level for all analyses was set to *P*<0.05. The expression was considered to display a daily rhythm if it had *P*<0.05 by both the one-way ANOVA and the cosinor analysis. Additionally, we used randomization techniques employed in REST 2009 [41] to test for significant differences in the expression of opsin genes among samples with different treatments.

### MIQE Guidelines

Herein, we followed the Minimum Information for Publication of Quantitative Real-Time PCR Experiments (MIQE) guidelines [42] to increase the reliability and the integrity of the results and to promote the effort for experimental consistency and transparency between research laboratories. A MIQE checklist was provided in Table S3.

## Results

### Amplification of Opsin Genes from *C. solmsi*

Four full-length opsin gene mRNA sequences including 3′UTR and 5′UTR were obtained from *C. solmsi* through RT-qPCR and RACE-PCR. Blast searches of GenBank determined that the inferred amino acid sequences were highly similar to opsin sequences of other insects [43]. The four translated sequences involved 1245 bp encoding 413 amino acids (aa), 1197 bp encoding 398 aa, 1176 bp encoding 391 aa, and 1125 bp encoding 374 bp, which corresponded to the LW1, LW2, blue, and UV opsin genes, respectively. In addition, splicing variants in the 3′UTRs of LW2 and UV opsin genes were detected in the product of the 3′ RACE and sequenced (Figure S1). Coding sequences of the four genes were deposited in GenBank under the accession numbers of JX402130–JX402133.

### Phylogenetic and Molecular Evolutionary Analyses

**Figure 1:**
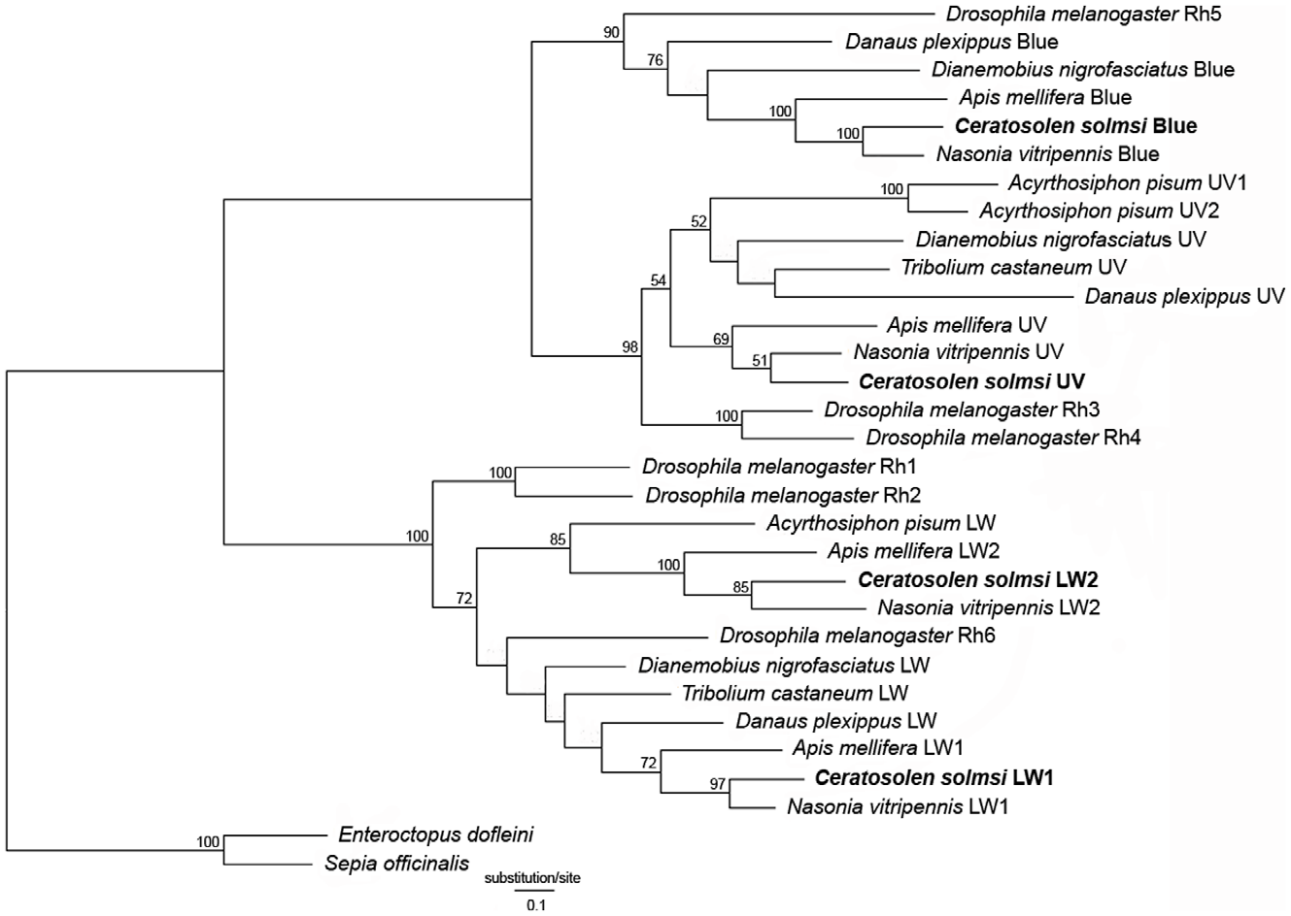
Phylogenetic position of opsin genes in *Ceratosolen solmsi*. The ML tree is constructed from full-length coding sequences of insect opsin genes. Bootstrap values >50 are shown at the nodes. Sequences obtained from *C. solmsi* are in bold.

Phylogenetic analysis of opsins from *C. solmsi* and other insects indicated that the fig wasp had the same repertoire of opsins as did *Apis mellifera* and *Nasonia vitripennis*. This involved two LW opsins, one blue opsin, and one UV opsin (Figure 1). Each class of opsin gene from *C. solmsi* clustered with the parasitic wasp *N. vitripennis*, which formed the sister-group of fig wasp opsins in our ML tree.

Theoretically, PAML analyses required the true relationships among genes. Most hymenopteran insects were shown to share the same sets of color receptors [2], [44] and we found them to have the same classes of opsin genes. The evolutionary history of all four opsin genes was expected to be concordant with the phylogeny of hymenopteran species but our gene trees (Figure S2, S3, S4, S5) were not so. Thus, we employed an independent species tree for hymenopteran insects (Figure S6) from previous studies [24], [45]–[48]. Branch lengths for each gene were estimated on the gene trees separately by using Codeml with the M0 model. Subsequently, these trees were used for ML estimates of selective forces in hymenopteran opsins. The M0 model resulted in an average ω of 0.05743 for LW1, 0.06902 for LW2, 0.05042 for blue, and 0.04998 for UV opsin genes, suggesting that all four genes underwent strong purifying selection (*P*<0.01) (Table S4, S5). In the site models, only three positively selected sites were detected for LW2 (*P<*0.05). We also repeated the tests based on the gene trees and obtained a similar result (data not shown).

**Figure 2:**
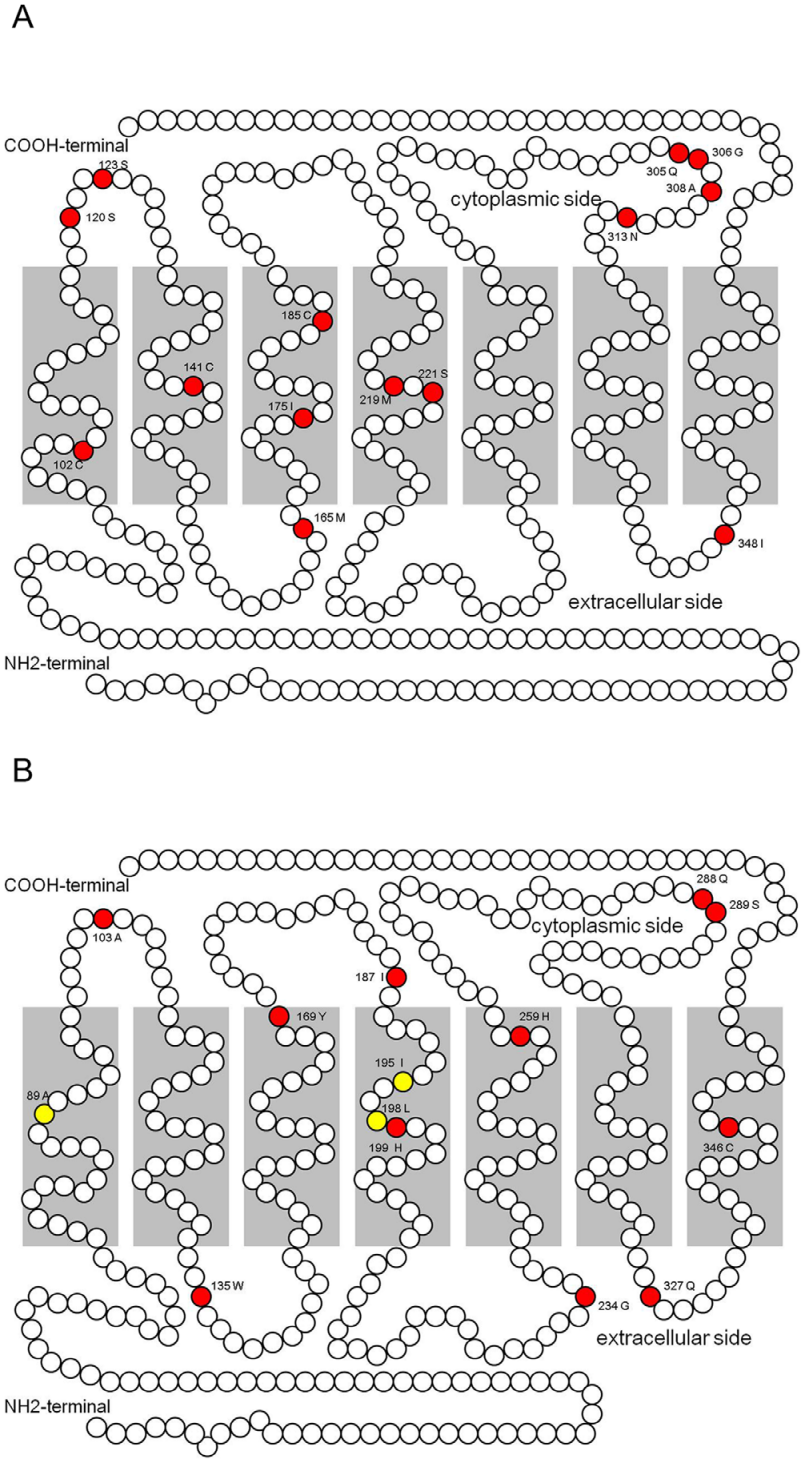
Mapping of positively selected sites on the secondary structure of opsin LW1 (A) and LW2 (B) in Ceratosolen solmsi. Positively selected sites determined with site models and branch-site models are highlighted in yellow and red, respectively.

Because *C. solmsi* and *Nasonia* had parasitic lifestyles, we also tested the selective pressure on the branch of their common ancestor (branch ‘a’ in Figure S6). The Branch model analysis indicated that UV opsin of branch ‘a’ was under purifying selection (*P*<0.001), whereas neutral evolution was not rejected for LW1, LW2, and blue opsin genes. Along branch ‘a’, 14 and 11 positively selected sites were indentified for LW1 and LW2, respectively upon implementation of the Branch-site model of selection. These sites were mapped on the secondary structure of the two genes for *C. solmsi*. Sites 123, 219, and 308 in LW1 opsin corresponded to sites 103, 199, and 289 in LW2, respectively (Figure 2), suggesting that LW1 and LW2 might have undergone similar selective pressure at some sites. No positively selected sites were detected for the UV opsin gene. Six positively selected sites were found in the blue opsin gene using the Branch-site model, whereas LRT could not reject neutral evolution of these sites (Table S5), suggesting that they were false positive sites [49].

### qPCR Assay Validations

Amplification efficiencies of the six primer pairs ranged from 91.2% to 105.7% as determined by standard curve analysis Correlation coefficients (R^2^) varied from 0.992 to 1.000 (Table S6). Melting curve analysis of RT-qPCR indicated single products for all six primer pairs (data not shown). These products were confirmed by sequencing. The possibility of amplification from reagent and genomic DNA contamination was eliminated with no-template and no-RT controls (data not shown).

### Effect of Light and Time on Opsin Gene Expression

Expression of all four opsin genes varied as a function of light, time, and light* time interaction in *C. solmsi*, except for the LW2 opsin gene in males (*F*_7,32_ = 1.676, *P* = 0.1505), which was not affected significantly by time (Table 1, 2). Considering the contribution to the total variation in opsin gene expression, the time factor had a stronger effect than light on blue and UV opsin gene expression in both sexes, and on LW2 opsin gene expression in females. However, the analyses also indicated a substantial effect of light on LW1 (accounted for 65.59% of total variation) and LW2 (accounted for 73.63% of total variation) opsin gene expression in males (Table 2).

**Table 1:** Univariate analyses of *opsin* expression in female *C. solmsi*.

Source ofvariation	df	MS	*P*	% of totalvariation
*LW1*:				
**Light**	**2**	**0.05963**	**<0.0001**	**35.53**
**Time**	**7**	**0.01558**	**<0.0001**	**32.49**
Light*Time	**14**	**6.315E-03**	**<0.0001**	**26.34**
**error**	**48**	**3.945E-04**		
*LW2*:				
**Light**	**2**	**5.293E-04**	**<0.0001**	**20.33**
**Time**	**7**	**2.919E-04**	**<0.0001**	**39.25**
Light*Time	**14**	**1.366E-04**	**<0.0001**	**36.73**
**error**	**48**	**3.998E-06**		
*Blue*:				
**Light**	**2**	**4.331E-06**	**<0.001**	**7.94**
**Time**	**7**	**6.862E-06**	**<0.0001**	**44.06**
Light*Time	**14**	**3.219E-06**	**<0.0001**	**41.33**
**error**	**48**	**1.516E-07**		
*UV*:				
**Light**	**2**	**1.926E-06**	**<0.0001**	**16.82**
**Time**	**7**	**1.430E-06**	**<0.0001**	**43.70**
Light*Time	**14**	**4.856E-07**	**<0.0001**	**29.68**
**error**	**48**	**4.679E-08**		

Notes: df = degrees of freedom, MS = mean- square, *P* = probability. Terms in bold indicate P<0.05.

**Table 2:** Univariate analyses of *opsin* expression in male *C. solmsi*.

Source ofvariation	df	MS	*P*	% of totalvariation
*LW1*:				
**Light**	**1**	**7.373E-03**	**<0.0001**	**65.59**
**Time**	**7**	**1.651E-04**	** = 0.0015**	**10.28**
Light*Time	**7**	**2.173E-04**	** = 0.0002**	**13.54**
**error**	**32**	**3.718E-05**		
*LW2*:				
**Light**	**1**	**1718**	**<0.0001**	**73.63**
Time	7	50.29	= 0.1505	1.624
Light*Time	**7**	**116.1**	** = 0.0037**	**3.749**
**Error**	**32**	**30.01**		
*Blue*:				
**Light**	**1**	**9.085E-04**	** = 0.0004**	**8.22**
**Time**	**7**	**5.466E-04**	**<0.0001**	**34.60**
Light*Time	**7**	**6.342E-04**	**<0.0001**	**40.15**
**Error**	**32**	**5.887E-05**		
*UV*:				
**Light**	**1**	**2.116**	** = 0.0037**	**9.67**
**Time**	**7**	**1.059**	** = 0.0008**	**33.90**
Light*Time	**7**	**0.7759**	** = 0.0058**	**24.83**
**Error**	**32**	**0.2160**		

Notes: df = degrees of freedom, MS = mean- square, *P* = probability. Terms in bold indicate P<0.05.

### Rhythmicity of Opsin Expression in *C. solmsi*

**Figure 3:**
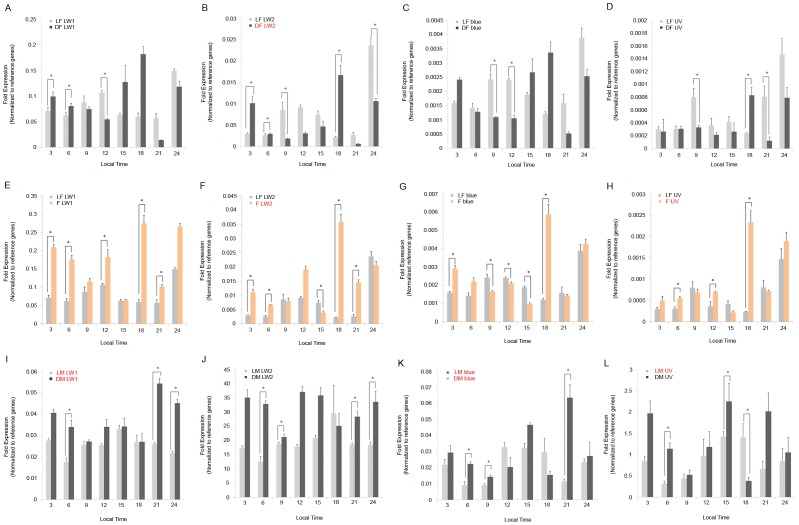
Relative expressions of the four opsin genes in female and male *Ceratosolen solmsi*. Abundance of mRNA in opsin genes relative to reference genes at each time point are represented by bars. Samples from inside of light (L) and dark (D) treated fig fruits are denoted by grey and black bars, respectively, and emerged females (F) exposed to light were denoted by orange bars. Significant differences in opsin gene expressions caused by light treatment at each time point are marked with an asterisk. The genes that show significant rhythmicity in a 24 hour cycle for each treatment are shown in red. Error bars (+ S.E.M for three biological replicates) are shown for all samples. LF: fig-female light; DF: fig-female dark; F: emerged female light; LM: fig-male light; DM: fig-male dark.

Based on one-way ANOVA and cosinor analyses, the following opsin genes were rhythmically expressed: LW2 (one-way ANOVA, *P*<0.0001; Cosinor, *P* = 0.0302) in females from dark treated figs; LW2 (one-way ANOVA, *P*<0.0001; Cosinor, *P* = 0.0191) and UV (one-way ANOVA, *P*<0.0001; Cosinor, *P* = 0.0257) in emerged females with light treatment. For males, the genes that showed rhythmic expression were as follows: LW1 (one-way ANOVA, *P* = 0.0006; Cosinor, *P* = 0.0018), blue (one-way ANOVA, *P* = 0.0004; Cosinor, *P* = 0.0049) and UV (one-way ANOVA, *P* = 0.0245; Cosinor, *P* = 0.0014) in individuals from light treated figs; and LW1 (one-way ANOVA, *P*<0.0001; Cosinor, *P* = 0.0021) and blue (one-way ANOVA, *P*<0.0001; Cosinor, *P* = 0.0328) in individuals from dark treated figs. Expression of all genes in females from light treated figs did not show significant rhythmicity (Table 3, Figure 3).Analyses of mRNA levels of opsin genes under different light conditions were normalized to reference genes. We then compared treatments involving light and no light. Levels of expressions for reference genes largely differed between females and males (data available on request). Thus, we did not compare the mRNA levels of opsin genes between sexes but rather evaluated the response of opsin gene expression levels to light within each sex. The levels of expression of the opsin genes oscillated in most samples in the 24 hour cycle, although not all of them showed rhythimicity. The only exception was LW2 of males from light treated figs, which showed no significant change of mRNA levels in a daily cycle (one-way ANOVA, *P* = 0.1505) (Table 3). The diurnal expression patterns of all four opsin genes were remarkably distinct. Females from fig fruits maintained in constant darkness had a stronger rhythmicity in opsin expression than did females in the light treated fruits. In general, opsin mRNA expression in females fluctuated with larger amplitude than in males (Figure 3).

**Table 3:** Statistical values of rhythmic expression of opsin genes in *C. solmsi*.

Gene	Significance of rhythmic expression (One-way ANOVA/Cosinor analysis)				
	LF	DF	F	LM	DM
*LW1*	***P*** **<0.0001/** *P* = 0.1232	***P*** **<0.0001/** *P* = 0.1911	***P*** **<0.0001/** *P* = 0.0849	***P*** ** = 0.0006/** ***P*** ** = 0.0018**	***P*** **<0.0001**/***P*** ** = 0.0021**
*LW2*	***P*** **<0.0001/** *P* = 0.2212	***P*** **<0.0001/** ***P*** ** = 0.0302**	***P*** **<0.0001/** ***P*** ** = 0.0191**	*P* = 0.1531/***P*** ** = 0.0253**	***P*** ** = 0.0061**/*P* = 0.9164
*Blue*	***P*** **<0.0001/** *P* = 0.1358	***P*** **<0.0001/** *P* = 0.0512	***P*** **<0.0001/** *P* = 0.0867	***P*** ** = 0.0004**/***P*** ** = 0.0049**	***P*** **<0.0001**/***P*** ** = 0.0328**
*UV*	***P*** **<0.0001/** *P* = 0.0611	***P*** ** = 0.0021/** *P* = 0.2326	***P*** **<0.0001/** ***P*** ** = 0.0257**	***P*** ** = 0.0245/** ***P*** ** = 0.0014**	***P*** ** = 0.0045/** *P* = 0.5136

Terms in bold indicate P<0.05. Genes with both *P* values <0.05 in One-way ANOVA and Cosinor analyses are considered rhythmically expressed.

To evaluate the effect of light on opsin gene expression, we made several comparisons for each sex (fig-females light vs. dark; fig-females light vs. emerged females light; fig-males light vs. dark).

Among females collected from figs in light and dark environments (fig-females light vs. dark; A–D in Figure 3), expressions of the four opsin genes were up- or down-regulated in samples collected in dark environments at different time-points. The significantly different time points were randomly distributed (A–D in Figure 3). Thus, we could not unambiguously determine if patterns of opsin gene expression differed between female wasps collected from figs with dark vs. light treatments.

The comparisons among the emerged females with light treatment and females from inside of light treated figs (emerged female light vs. fig-females light; E–H in Figure 3) detected significant up-regulation for all genes at 18:00 in the former group. Up-regulation was also oberved for LW1 at the time points 3:00 (*P*<0.001), 6:00 (*P*<0.001), 12:00 (*P* = 0.033), 18:00 (*P*<0.001), and 21:00 (*P* = 0.021) and for UV at 6:00 (*P* = 0.045), 12:00 (*P* = 0.033), and 18:00 (*P* = 0.024). The LW2 and blue opsin gene seemed to follow the pattern of up-regulation at all or a subset of 3:00, 6:00 and 18:00, although down-regulation was also detected at some other time points (Figure 3E–H).

Comparisons of the males collected from light and dark treated figs (fig-males light vs. dark; I–L in Figure 3) found significant differences. LW1 was up-regulated in males collected from figs maintained in the dark at time points of 6:00 (*P*<0.001), 21:00 (*P* = 0.013), and 24:00 (*P*<0.001). The expressions of LW2 and blue were similar, with up-regulation at 6:00 (LW2, *P*<0.001; blue, *P*<0.001), 9:00 (LW2, *P* = 0.021; blue, *P* = 0.012), 21:00 (LW2, *P* = 0.020; blue, *P* = 0.022), and 24:00 (LW2, *P* = 0.045). Down-regulation occurred only in UV opsin gene expression at 18:00 (*P* = 0.020) in the samples from figs maintained in darkness, while up-regulated at 6:00 (*P* = 0.045) and 15:00 (*P* = 0.025) (I–L in Figure 3).

### Relative Proportion of Opsin Gene Expression

**Figure 4:**
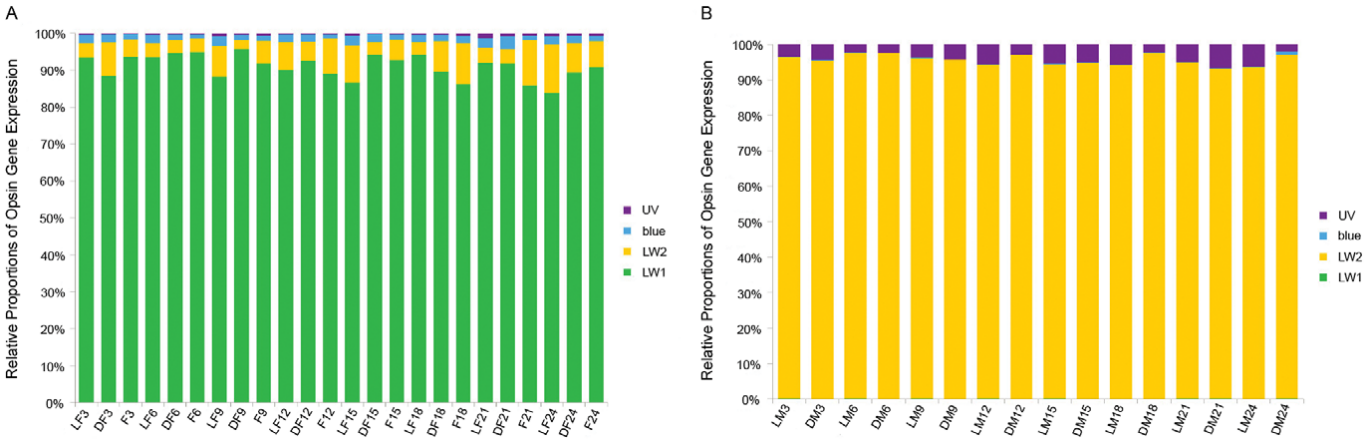
Relative proportion of the opsin gene expression in female (A) and male (B) *Ceratosolen solmsi*. Relative proportion of expression for each gene is indicated by a different color.

We compared the expression of each of the four opsin genes relative to the total opsin gene expression. The patterns were quite different between females and males but similar within each gender. All female samples were as follows: LW1> LW2> blue>UV, with LW1 having the highest relative proportion of expression (proportion >80%). In contrast, male samples had a different pattern: LW2> UV>blue & LW1; LW2 occupied >90% of the expressed transcripts and expression levels of blue and LW1 opsins were minute. The relative proportions of opsin gene expression had no dramatic diel changes in either gender (Figure 4).

## Discussion

### Heterogeneous Selective Pressure on Opsin Genes in Hymenoptera and Chalcidoidea

Hymenopterans occur in a variety of habitats yet the species show surprisingly little variation in spectral sensitivity. Most hymenopterans are trichromatic, possessing UV, blue, and green receptors [44]. *Ceratosolen solmsi* also has the same composition of functional opsin genes as bees.

The LW2 opsin gene, which only occurs in Hymenoptera, appears to represent an ancient paralog of LW1 opsin genes [50]. In *Apis mellifera*, the LW1 opsin gene (*AmLop1*) is expressed in the compound eye, whereas the LW2 opsin gene (*AmLop2*) is ocellus-specific [51]. Differential expression in ocelli and compound eyes of multiple LW opsin genes also occurs in the fruitfly *Drosophila melanogaster* [52] and the cricket *Gryllus bimaculatus* [53]. Our results indicate that LW1 has a lower ω value than LW2 (*P* = 0.015) in the analysis of selective pressure when applying the M0 (one-ratio) model. Compound eyes and ocelli have different functions, thus, different selective pressure should have acted on each LW opsin paralog. In particular, female fig pollinators have normal ocelli but males lack these organs. Since the LW2 opsin gene is strongly expressed in the males (Figure 4B), it is likely that LW2 has undergone a neofunctionalization within fig pollinators. The higher nonsynonymous substitution rate in the LW2 opsin gene might faciliate this process.

Our analyses also discover that LW opsin genes have higher ω value than blue and UV in hymenopterans, suggesting that they underwent relatively strong selective pressure. After the gene duplication, several amino acid sites in LW1 and LW2 opsins experienced shifts in selective constraints [50]. Even using the rigid site-models, we detect three sites with ω>1 for LW2, indicating that natural selection affected this gene in hymenopterans. Our Branch-site model analysis detects a series of positively selected sites of these genes in the Chalcidoidea. This suggests that natural selection also contributed to evolution of both LW1 and LW2 after emergence of ancestral chalcidoids, although no experimental evidence exists for functional advantages of these sites.

The Branch-site model identifies six positively selected sites for the blue opsin gene along branch ‘a’. However, LRT between the branch-site model and its null model cannot reject the possibility of neutral evolution for this gene. Therefore, high dN/dS at these sites must have resulted from relaxed selectional constraint [49]. Considering the low level of expression for the blue opsin gene in both female and male pollinators, we speculate that this gene experienced weaker selective pressure than the LW opsin genes.

The branch model analyses identify the UV opsin gene as being under purifying selection on branch ‘a’ (*P*<0.001). The ancestral arthropod most probably had an UV opsin gene [54] and so far no loss of UV sensitivity has been found in any insect species with functional eyes [55]. Instead, duplications of UV opsin genes are reported for several lineages of insects including fruit flies [56], beetles [57], and butterflies [13]. Color sensitivity in the UV plays an important role in navigation in both flying and terrestrial invertebrate animals [58]. Although male fig pollinators live their entire lives inside the body of their host, and we cannot determine if selective pressure acts on their UV opsin genes. This gene must have experienced strong purifying selection in the females, which need to fly for searching another host fig fruit after emerging from the syconia. Notwithstanding, we cannot exclude the possibility that the UV opsin gene has an alternative function and is not used for vision.

### Expression of Opsin Genes in Fig Pollinators

Both light and time can affect opsin gene expression in female and male *C. solmsi*, although effects of the two factors are not equal for the four genes. Surprisingly, light most likely influences the expression of LW1 and LW2 opsin genes in males. This result suggested that although male pollinators live all their lives inside the fig fruits [22], [23], visible light still plays an important part in the regulation of their opsin gene expression. In females, the time effect on LW2 opsin gene expression outpaces the light effect (Table 1), Time does not significantly affect expression of this gene in males. LW2 is the only gene that does not show rhythmic expression in male samples from both light and dark treated figs. However, females in dark treated figs tend to express this gene rhythmically. Regardless, males have an extremely high proportion of LW2 opsin gene expression (Figure 4). Further, the difference in expression rhythmicity of the LW2 opsin gene is also sexually dimorphic.

Generally, males in the light treated figs exhibit stronger rhythmicity of opsin gene expression than those in dark treated figs. The rhythmicity of UV opsin gene expression disappears when the figs are dark treated. In contrast, expression of all genes in females inside syconia shows no rhythmicity, except LW2 opsin gene in dark treated figs. When females emerge from fig fruits, they tend to express LW2 and UV opsin genes rhythmically. No comparison among groups of males exists because males that emerge from the syconia die shortly thereafter, a comparison would be biologically unrealistic.

The comparisons of males collected from figs in light and dark present unexpected results. LW1, LW2 and blue opsin genes are up-regulated when figs are put in darkness and we cannot detect this effect in females. This suggests that the expression of opsin genes in males shows a different response to light than in females. Expression levels of LW2 (about 10–40 times than the reference genes) in male samples are particularly higher than the other genes (Figure 3J), which indicates that expression of LW2 opsin gene is biased in males pollinators. The lifecycles of fig pollinators are intimately synchronized with the development of their hosts [23]. One major morphological difference between female and male fig pollinators is that mature females develop compound eyes. In contrast, males have degenerate eyes and remain inside the syconia throughout their lives. Further experimentation is necessary to test whether the males and the females have different spectral sensitivities.

Both the light and time factors can affect the expression of opsin genes in fig pollinators, and light shows both up- and down- regulatory effects over time. This suggests that an endogenous pacemaker also controls the daily levels of opsin mRNA in fig pollinators and light resets it. The Opsin gene expression of both females and males inside the syconia was affected by light, although it remains unknown if fig pollinators in fig fruits employ functional vision. Light-sensing in fig fruits might be necessary for fig pollinators to synchronize their activity with the lifecycle of their host [22], [23]. Levels of opsin mRNA have been suggested to be regulated by light in a species-specific manner [59], [60]. Our study reveals that the light factor and the time factor can have differently affect the opsin gene expression in female and male fig pollinators. Long-term co-evolution with figs might drive this pattern.

The relative proportions of expression for the four opsin genes differ dramatically between female and male fig pollinators. Females have the highest expression of LW1, and together LW1 and LW2 constitute most of the opsin expression. In contrast, males most strongly express LW2 followed by UV opsin. All specimens are mature wasps. Females must emerge from the enclosed syconia into an environment where a wide spectral range of visible light exists, and this might correspond with the high expression of both LW opsin genes. Although the relative expression of opsin genes differ significantly between female and male pollinators, further work is necessary to test if these differences are a consequence of contrasting lifestyles.

Deep phylogenies imply a long-term co-evolutionary history for figs and their pollinator wasps [61]. The evolution and expressional plasticity of opsin genes in *C. solmsi* have corresponding evolutionary implications. Accompanied by phenotypic specializations, co-evolution history with figs might have influenced evolution and expression plasticity of opsin genes in fig pollinators.

## Supporting Information

Figure S1 **Variable splicing in 3′UTR of of LW2 and UV opsin gene in** ***Ceratosolen solmsi*** **.** DNA marker: Trans2k (Transgene, China).
(TIF)

Figure S2 **The maximum likelihood tree for the LW1 opsin genes of Hymenoptera using sequence from the cricket (** ***Dianemobius nigroufasciatus*** **) as outgroup.** Scale bar represents substitution per site.
(TIF)

Figure S3 **The maximum likelihood tree for the LW2 opsin genes of Hymenoptera using sequence from the cricket (** ***Dianemobius nigroufasciatus*** **) as outgroup.** Scale bar represents substitution per site.
(TIF)

Figure S4 **The maximum likelihood tree for the blue opsin genes of Hymenoptera using sequence from the cricket (** ***Dianemobius nigroufasciatus*** **) as outgroup.** Scale bar represents substitution per site.
(TIF)

Figure S5 **The maximum likelihood tree for the UV opsin genes of Hymenoptera using sequence from the cricket (** ***Dianemobius nigroufasciatus*** **) as outgroup.** Scale bar represents substitution per site.
(TIF)

Figure S6 **Phylogeny used for selection analysis in the present study, the branch leading to parasitoid wasps are labelled as ‘a’.** This is a deduced species tree based on previous studies (Astruc et al., 2004; Moreau et al., 2006; Munro et al., 2001).
(TIF)

Table S1 **Primers used for amplification of full length mRNA of opsin genes in** ***Ceratosolen solmsi.***
(DOC)

Table S2 **Accession numbers of opsin genes in this study.**
(DOC)

Table S3 **MIQE checklist.**
(DOC)

Table S4 **Likelihood values and parameters estimated by codeml for fig pollinator opsin genes.**
(DOC)

Table S5 **Likelihood ratio tests (LRTs) for positive selection of fig pollinator opsin genes.**
(DOC)

Table S6 **Descriptions of primer pairs used for RT-qPCR analysis.**
(DOC)
