# Supplementary figures and images for: Evolution and Expression Plasticity of Opsin Genes in a Fig Pollinator, Ceratosolen solmsi
Source: PLoS One. 2013 Jan 16;8(1):e53907. doi: 10.1371/journal.pone.0053907 (PMC3547053; doi:10.1371/journal.pone.0053907)

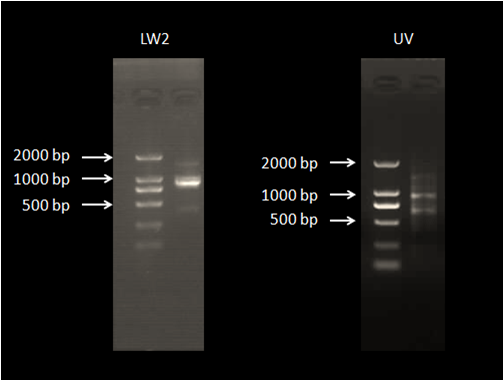

Supplement: Figure S1 — Variable splicing in 3′UTR of of LW2 and UV opsin gene in Ceratosolen solmsi . DNA marker: Trans2k (Transgene, China). (TIF) [file pone.0053907.s001.tif]

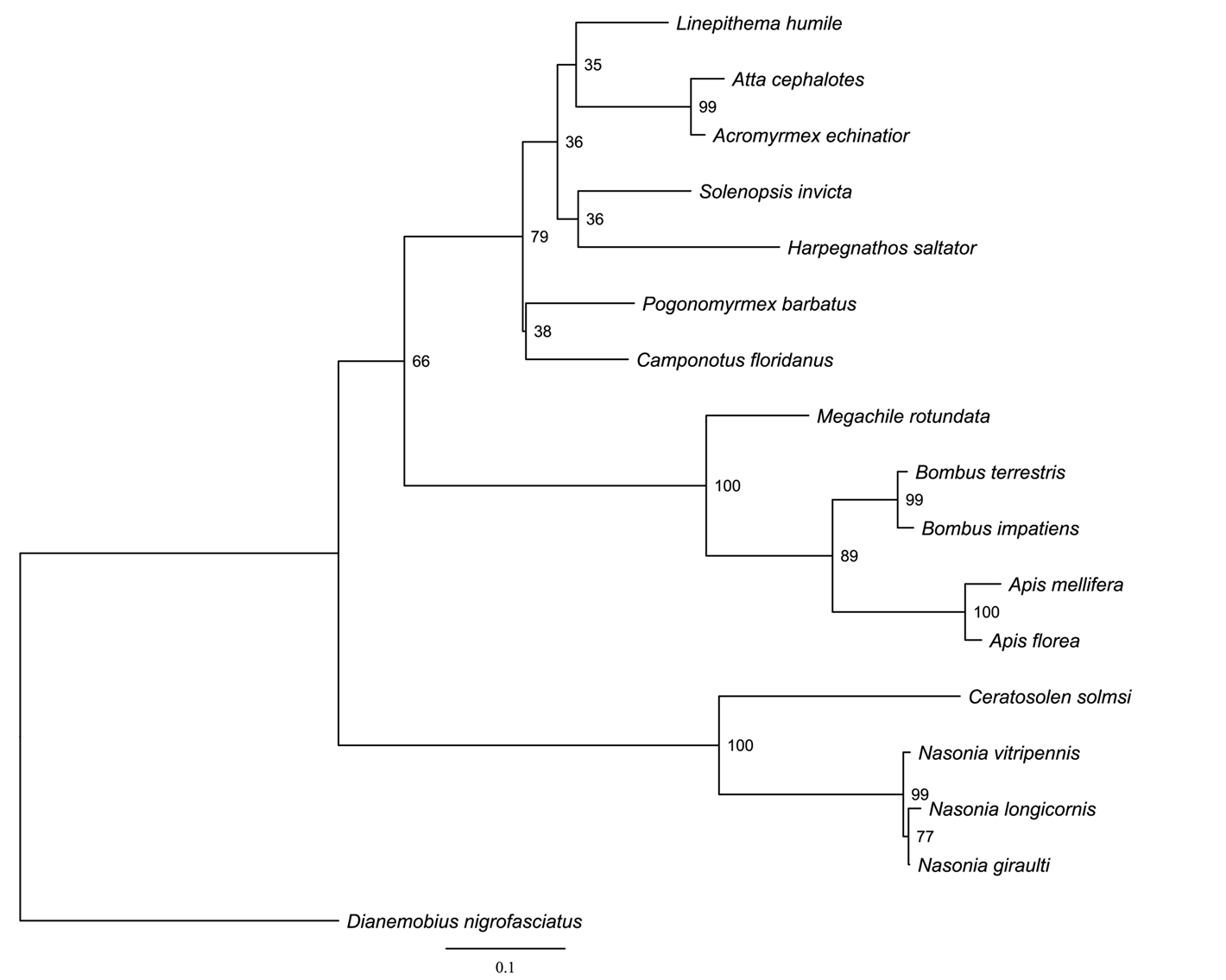

Supplement: Figure S2 — The maximum likelihood tree for the LW1 opsin genes of Hymenoptera using sequence from the cricket ( Dianemobius nigroufasciatus ) as outgroup. Scale bar represents substitution per site. (TIF) [file pone.0053907.s002.tif]

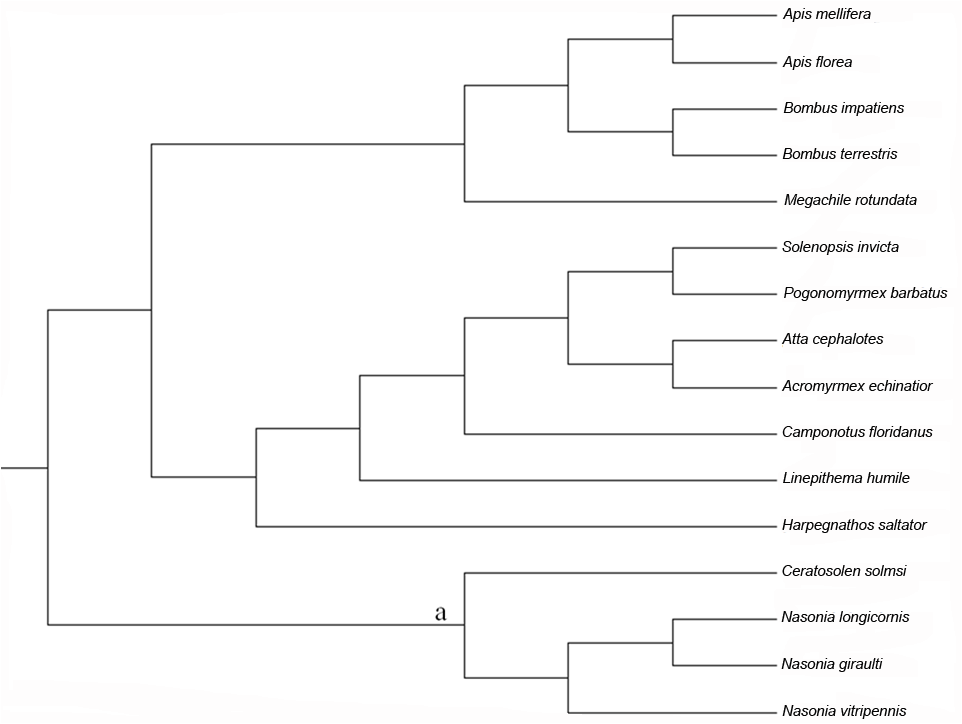

Supplement: Figure S6 — Phylogeny used for selection analysis in the present study, the branch leading to parasitoid wasps are labelled as ‘a’. This is a deduced species tree based on previous studies (Astruc et al., 2004; Moreau et al., 2006; Munro et al., 2001). (TIF) [file pone.0053907.s006.tif]
