# Supplementary material for: Evolution and Expression Plasticity of Opsin Genes in a Fig Pollinator, Ceratosolen solmsi
Source: PLoS One. 2013 Jan 16;8(1):e53907. doi: 10.1371/journal.pone.0053907 (PMC3547053; doi:10.1371/journal.pone.0053907)
Supplement: Table S1 — Primers used for amplification of full length mRNA of opsin genes in Ceratosolen solmsi. (DOC) [file pone.0053907.s007.doc]

**Table S1. Primers used for amplification of full length mRNA of opsin genes in *Ceratosolen solmsi*.**

| Primer Names | Primer Sequences |
| --- | --- |
| Lop1f2 | 5’-GACAARGYKCCRCCYGAKAT-3’ |
| Lop1r1 | 5’-GCYCGRTAYTTSGGATGACT-3’ |
| Lop1r2 | 5’-CCRTARACKATCGGRTTGT-3’ |
| Fwbof | 5’-GYTGCGTCRTATGGATATT-3’ |
| Fwbor | 5’- CTCGTGAATKCCCATCC-3’ |
| Bf | 5’-ACGGYTGCGTCGTWTGGA-3’ |
| Br | 5’-CGTAKACCCARGGATCRATGC-3’ |
| UVOF1 | 5’-CAYATACCSGARCAYTGGC-3’ |
| UVOF2 | 5’-GTMATMTGGATMTTYTGCGC-3’ |
| UVOF3 | 5’-TCACKCCRTABGGWGTCC-3’ |
| UVOF4 | 5’-CTKATRGCRTABACRTABGG-3’ |
| CSML1F1 | 5’-ATGTAATCGTCAAGGGTCTGTCGGCTAA-3’’ |
| CSML1F2 | 5’-GCACCTTTGTTTGGCTGGAATCGTT-3’ |
| CSML1R1 | 5’-TTAGCCGACAGACCCTTGACGATTACAT-3’ |
| CSML1R2 | 5’-CAGAAAAGGCGAGGTTGATGACCAGAA-3’ |
| CSML2F1 | 5’-TGGTTGGGCTGGCAATGGAGTTGTAGT-3’ |
| CSML2F2 | 5’-GTTCCTTATGCGGCGGTGCCTCAATA-3’ |
| CSML2R1 | 5’-TTCCCAGCCATTCATCCGATACATAGTCAG-3’ |
| CSML2R2 | 5’-TATTGAGGCACCGCCGCATAAGGAAC-3’ |
| CSMBF1 | 5’-CAATCTTCTTTCTATTTGTTTGTGCCTGGAC-3’ |
| CSMBF2 | 5’-TTTCTATTTGTTTGTGCCTGGACACCG-3’ |
| CSMBR1 | 5’-GATTGTAAAGGCAACTTTGGCTATTCTCATCT-3’ |
| CSMBR2 | 5’-GGCAGAATAGTGAAAGGCATCGACCAGA-3’ |
| CSMUVF1 | 5’-ATCTTCACCTTCTCCTACGCCATTCCC-3’ |
| CSMUVF2 | 5’-AAGCAACCAGAACCAGCAGACCCAATC-3’ |
| CSMUVR1 | 5’-TGGGTCTGCTGGTTCTGGTTGCTTCTT-3’ |
| CSMUVR2 | 5’-GTCTCGCTGGAGTCGGTGAGGTAGTCG-3’ |
