# Supplementary material for: Evolution and Expression Plasticity of Opsin Genes in a Fig Pollinator, Ceratosolen solmsi
Source: PLoS One. 2013 Jan 16;8(1):e53907. doi: 10.1371/journal.pone.0053907 (PMC3547053; doi:10.1371/journal.pone.0053907)
Supplement: Table S2 — Accession numbers of opsin genes in this study. (DOC) [file pone.0053907.s008.doc]

**Table S2 Accession numbers of opsin genes in this study.**

| Species | LW1 opsin gene | LW2 opsin gene | Blue opsin gene | UV opsin gene |
| --- | --- | --- | --- | --- |
| *Sepia offcinalis* | AF000947 | - | - | - |
| *Octopus dofleini* | X07797.1 | - | - | - |
| *Acyrthosiphon pisum* | XM_001947695.2 | - | - | XM_001951553.2  XM_001951578.2 |
| *Dianemobius nigrofasciatus* | FJ232921 | - | AB291232.1 | AB458852.1 |
| *Apis mellifera* | NM_001011639.2 | NM_001077825. | NM_001011606.1 | NM_001011605.1 |
| *Nasonia vitripennis* | NM_001170908.1 | XM_003427424.1 | XM_001604572.2 | XM_001608024.2 |
| *Tribolium castaneum* | NM_001162519 | - | - | XM_965251.1 |
| *Drosophila melanogaster* | NM_079683  NM_079674  Z86118 | - | NM_057748.4 | M17718.1  NM_057353.3 |
| *Danaus plexippus* | AY605545.1 | - | AY605544.1 | AY605546.1 |
| *Megachile rotundata* | AFJA01007060.1: 16899-17219; 17408-17655; 17726-17984; 18046-18212; 18312-18438 | AFJA01007060.1: 20555-20739; 20234-20472; 20057-20163; 19890-20002; 19681-19828; 19493-19603; 19270-19436; 19102-19216 | AFJA01003332.1: 17719-17780; 17324-17515; 16915-17130; 16723-16853; 16462-16634; 16100-16257; 15942-16037; 15725-15839 | AFJA01000488.1: 27236-27459; 27771-27986; 28172-28553; 28672-28856; 28955-29064 |
| *Apis florea* | AEKZ01003979.1: 35639-35961; 36075-36322; 36417-36675; 36769-36935; 37030-37162 | AEKZ01003979.1: 39535-39704; 39222-39460; 39024-39130; 38842-38948; 38581-38728; 38371-38481; 38044-38210; 37862-37970 | AEKZ01007958.1: 2979-3037; 2603-2794; 2211-2426; 2011-2141; 1757-1930; 1474-1631; 1303-1400; 1091-1196; | AEKZ01009465.1: 24979-25202; 24588-24803; 23727-24108; 22979-23163; 22435-22543 |
| *Bombus impatiens* | XM_003486900.1 | [XM_003486899.1](http://www.ncbi.nlm.nih.gov/nucleotide/350403910?report=genbank&log$=nucltop&blast_rank=2&RID=NZYBFAAZ01N) | XM_003494875.1 | XM_003491095.1 |
| *Bombus terrestris* | XM_003401011.1 | [XM_003401007.1](http://www.ncbi.nlm.nih.gov/nucleotide/340725393?report=genbank&log$=nucltop&blast_rank=1&RID=NZYBFAAZ01N) | XM_003400337.1 | XM_003402514.1 |
| *Nasonia giraulti* | ADAO01102587.1: 8-283; ADAO01102586.1: 1050-1297; 722-974; 485-651; 262-406 | ADAO01102581.1: 7810-7873; 7960-8037; 8111-8193; 8270-8443; 8524-8721; 9365-9588; 9675-9913; 9997-10103; 10173-10279; 10356-10500; ADAO01102582.1: 5-118; ADAO01102583.1: 6-172; 264-393 | ADAO01172241.1: 117-295; 618-833; 1060-1190; 1261-1433; 1500-1654; 1716-1811; 1878-2004 | ADAO01293119.1: 3676-3923; 3012-3227; 2554-2935; 2264-2448; 2092-2194 |
| *Nasonia longicornis* | ADAP01017678.1: 17-286; ADAP01017677.1: 959-1206; 631-883; 394-560; 171-315 | ADAP01017669.1: 7825-7888; 7975-8052; 8126-8208; 8285-8458; 8539-8736; 9380-9603; 9695-9933; 10000-10106; 10178-10284; ADAP01017670.1: 4-157; ADAP01017671.1: 4-88; ADAP01017672.1: 1-159; 247-378 | ADAP01022982.1: 108-286; 609-824; ADAP01022983.1: 204-334; 405-577; 644-798; 860-955; 1022-1148 | ADAP01005783.1: 296-543; ADAP01005782.1: 3024-3239; 2566-2947; 2276-2460; 2104-2206 |
| *Acromyrmex echinatior* | AEVX01011144.1: 18725-19054; 18385-18632; 18042-18300; 17796-17962; 17537-17666 | AEVX01011144.1: 14109-14296; 14368-14606; 14708-14819; 14883-14989; 15085-15232; 15306-15416; 15533-15699; 15784-15904 | AEVX01012537.1: 17230-17291; 15666-15857; 14653-14848; 14235-14364; 13683-13855; 13428-13585; 13256-13351; 12469-12577 | AEVX01009625.1: 132286-132509; 132767-132982; 133599-133980; 134545-134729; 135689-135788 |
| *Atta cephalotes* | ADTU01020829.1: 15547-15876; 15979-16226; 16310-16568; 16645-16811; 16944-17073 | ADTU01020830.1: 119-236; 307-479; 586-695; 768-917; 1010-1115; 1187-1293; 1395-1632; 1708-1891 | ADTU01031203.1: 50-111; ADTU01025579.1: 1497-1688; 535-748; 124-254; ADTU01025580.1: 5057-5229; 4794-4551; 4620-4715; 3824-3932 | ADTU01002850.1: 588-842; 1096-1311; 1930-2311; 2850-3034; 4064-4163 |
| *Harpegnathos saltator* | AEAC01022378.1: 14723-15065; 14398-14647; 14059-14321; 13327-13498; 12430- 12567 | AEAC01022378.1: 4931-5124; 5255-5493; 5855- 5961; 6034-6140; 6309-6456; 6531-6641; 6967-7133; 7218-7338 | AEAC01015224.1: 39663-39721; 41455-41646; 42206-42421; 42712-42842; 43145-43317; 43401-43558; 43641-43736; 44104-44212 | AEAC01003716.1: 67307-67530; 67651-67866; 67989-68370;68482-68666; 68748-68850 |
| *Linepithema humile* | ADOQ01001409.1: 6071-6392; 5736-5983; 5395-5653; 5156-5322; 4915-5044 | ADOQ01001409.1: 1982-2175; 2256-2494; 2673-2779; 2855-2961; 3045-3192; 3266-3376; 3454-3620; 3706-3829 | ADOQ01008747.1: 33872-33933; 32288-32479; 31538-31753; 31119-31249; 30746-30919; 30471-30627; 30303-30398; 29930-30038 | ADOQ01008054.1: 11531-11754;10827-11042; 9835-10216; 9335-9519; 8935-9037 |
| *Camponotus floridanus* | AEAB01016114.1: 22248-22580; 21912-22159; 21550-21808; 21292-21458; 21076-21205 | AEAB01016114.1: 18170-18363; 18449-18687; 18806-18912; 18984-19090; 19161-19308; 19388-19498; 19672-19838; 19918-20038 | AEAB01031758.1: 55565-55626; 56959-57150; 57667-57882; 58125-58255; 58657-58829; 58931-59088; 59178-59273; 59441-59549 | AEAB01004928.1: 9117-9340; 9590-9805; 9961-10342; 10603-10787; 10870-10972 |
| *Pogonomyrmex barbatus* | ADIH01017695.1: 9456-9785; 9864-10111; 10201-10459; 10541-10707; 10836-10963 | ADIH01022477.1: 515-708; 799-1037; 1137-1243; 1321-1427; 1536-1683; 1760-1870; 1990-2156; 2232-2346 | ADIH01027094.1: 20577-20638; 22032-22223; 22954-23169; 23418-23548; 24434-24606; 24589-24846; 24926-25021; 25219-25327 | ADIH01029848.1: 2466-2689; 2973-3188; 3713-4094; 4516-4700; 5906-6008 |
| *Solenopsis invicta* | AEAQ01016032.1: 27177-27506; 25868-26115; 25518-25776; 21730-21896; 17528-17660 | AEAQ01016032.1: 8370-8564; 9415-9653; 10037-10143; 11260-11366; 12971-13118; 13190-13330; 13410-13576; 16438-16561 | AEAQ01002048.1: 85164-85225; 86610-86801; 87729-87944; 88222-88352; 88662-88834; 88931-89088; 89160-89255; 89411-89519 | AEAQ01061141.1: 585-808; AEAQ01016812.1: 8650-8865; AEAQ01016817.1: 1736-2117; AEAQ01023816.1: 387-571; AEAQ01023829.1: 17258-17360 |
