# Supplementary material for: Evolution and Expression Plasticity of Opsin Genes in a Fig Pollinator, Ceratosolen solmsi
Source: PLoS One. 2013 Jan 16;8(1):e53907. doi: 10.1371/journal.pone.0053907 (PMC3547053; doi:10.1371/journal.pone.0053907)
Supplement: Table S3 — MIQE checklist. (DOC) [file pone.0053907.s009.doc]

**Table S3 MIQE checklist.**

| **Item to check** | **Importance** | | **Details** |
| --- | --- | --- | --- |
| **Sample** | |  |  |
| **Description** | | E | Pollinators (*Ceratosolen solmsi*) of *Ficus hispida* |
| **Processing procedure** | | E | Female and male fig pollinators were light/ darked treated and collected from fig fruits |
| **If frozen, how and how quickly** | | E | Insect samples were immediately frozen in liquid nitrogen after they were collected |
| **If fixed, with what and how quickly?** | | E | Stored in sample Protector (TAKARA, China) immediately after frozen |
| **Sample storage conditions and duration** | | E | Samples were held at -20 ^o^C for less than a week before RNA isolation |
| **Experimental design** | |  |  |
| **Definition of experimental and control groups** | | E | No relative quantification were involved in this work, thus no control groups were defined |
| **Number within each group** | | E | 3 |
| **Nucleic acid extraction** | |  |  |
| **Procedure and/or instrumentation** | | E | For each RNA sample, total RNA of 40 individuals was extracted by using an EasyPureTM RNA kit (Transgen, China) |
| **Name of kit and details of any modifications** | | E | EasyPureTM RNA kit (Transgen, China). We exactly followed the protocols of the kit |
| **Details of DNase or RNase treatment** | | E | Genomic DNA was removed by treating with DNase I according to the standard protocols |
| **Contamination assessment (DNA or RNA)** | | E | No reverse transcription control (NRC) was performed for each RNA sample to assess the absence of DNA. |
| **Nucleic acid quantification** | | E | RNA concentration was determined by measuring the abosorbance at 260nm UV light |
| **Instrument and method** | | E | NanoDrop-2000 Spectrophotometer (Thermo, USA) |
| **RNA integrity: method/instrument** | | E | RNA integrity was assessed by electrophoresis on 1.0% agarose gels stained with ethidium bromide |
| **RIN/RQI or C_q_ OF 3’ and 5’ transcripts** | | E | N/A |
| **Inhibition testing (C_q_ dilutions, spike, or other)** | | E | Standard curve analyses were sufficient to test inhibition |
| **Reverse transcription** | |  |  |
| **Complete reaction conditions** | | E | TransScript II First-Strand cDNA Synthesis SuperMix (Transgen, China) was used to generate single-stranded cDNA total RNA with oligo-dT. For each sample, a template for no RT-control was prepared. |
| **Amount of RNA and reaction volume** | | E | Amount of RNA: 1μg; Reaction volume: 20μl |
| **Priming oligonucleotide and concentration** | | E | oligo-dT: 2μM |
| **Temperature and time** | | E | 50 ^o^C for 1 hour |
| **qPCR protocol** | |  |  |
| **Complete reaction conditions** | | E | PCR reactions were performed in a Mx3000P Real Time Thermocycler (Stratagene). A 20 μl PCR mixture was prepared containing 1 μl of template, 10μl TransStart Green qPCR SuperMix UDG(2x) (Transgen, China), 0.4μl Passive Reference Dye II(50x) (Transgen, China), 0.8μl primer mix(0.2μM), and 7.8 μl sterile water. The following thermal conditions for qRT-qPCR were used: 50^o^C for 2 min, 95^o^C for 10 min, and then the follwing: 95^o^C for 10 s, 54^o^C for 15 s and 72^o^C for 10 s for 40 cycles |
| **Reaction volume and amount of cDNA/DNA** | | E | Reaction volume: 20μl; amount of cDNA: 1μl per reaction volume |
| **Primer, (probe), Mg2, and dNTP concentrations** | | E | 500nM primers; 3mM MgCl_2_ ; 0.2 mM dNTP |
| **Polymerase identity and concentration** | | E | TransStart Green qPCR SuperMix UDG (2x) (Transgen, China) |
| **Buffer/kit identity and manufacturer** | | E | TransStart Green qPCR SuperMix UDG (2x) (Transgen, China) |
| **Additives (SYBR Green I, DMSO, and so forth)** | | E | Passive Reference Dye II(50x) (Transgen, China) |
| **Complete thermocycling parameters** | | E | 50^o^C for 2 min, 95^o^C for 10 min, and then the follwing: 95^o^C for 10 s, 54^o^C for 15 s and 72^o^C for 10 s for 40 cycles |
| **Specificity (gel, sequence, melt, or digest)** | | E | Melting curve analysis, gel electrophoresis and sequencing |
| **For SYBR Green I, C_q_ of the NTC** | | E | The signal of the amplification plot was late (C_q_>30), difference of C_q_ between NTC controls and cDNA samples was large |
| **Calibration curves with slope and y intercept** | | E | -3.58~-3.10 |
| **PCR efficency calculated from slope** | | E | Table1 of Paper |
| **R^2^ of calibration curve** | | E | Table1 of paper |
| **Linear dynamic range** | | E |  |
| **C_q_ variation at LOD** | | E |  |
| **Evidence for LOD** | | E | C_q_<35 for all samples |
| **If multiplex efficiency and LOD OF each assay** | | E | N/A |
| **qPCR analysis analysis program (sourse, version)** | | E | MxPro QPCR Software |
| **Method of C_q_ determination** | | E | Cq values were determined using threshold, which is determined using the Amplification-based Threshold method |
| **Outlier identification and disposition** | | E | None of Cq values was discarded |
| **Results for NTCs** | | E | The signal of the amplification plot was very late (C_q_>35) |
| **Justification of number and choice of reference genes** | | E | *UBC*; *RPL13a* |
| **Description of normalization method** | | E | N/A |
| **Number and stage (RT or qPCR) OF technical replicates** | | E | Duplicate |
| **Number and concordance of biological replicates** | | E | Triplicate |
| **Repeatability (intraassay variation)** | | E | ∆Cq < 0.5 for all duplicates |
| **Statistical methods for results significnce** | | E | Randomization techniques employed in REST 2009 |
| **Software (source, version)** | | E | REST2009 |
| **qPCR target information** | |  |  |
| **Gene symbol** | | E | Text |
| **Sequence accession number** | | E |  |
| **Amplicon length** | | E | Table 1 of paper |
| **In silico specificity screen (BLAST, and so on)** | | E |  |
| **Location of each primer by exon or intron (if applicable)** | | E | Primers were designed spanning the splicing sites |
| **What splice variants are targeted** | | E | N/A |
