# Supplementary material for: Evolution and Expression Plasticity of Opsin Genes in a Fig Pollinator, Ceratosolen solmsi
Source: PLoS One. 2013 Jan 16;8(1):e53907. doi: 10.1371/journal.pone.0053907 (PMC3547053; doi:10.1371/journal.pone.0053907)
Supplement: Table S4 — Likelihood values and parameters estimated by codeml for fig pollinator opsin genes. (DOC) [file pone.0053907.s010.doc]

**Table S4 Likelihood values and parameters estimated by codeml for fig pollinator opsin genes.**

| Models | P | LnL | Parameters | Positive selected sites |
| --- | --- | --- | --- | --- |
| LW1 opsin gene |  |  |  |  |
| One-ratio (LW1) | 32 | -6253.397834 | ω=0.05743 | None |
| M1a | 33 | -6186.240980 | p0=0.93160, p1=0.06840, ω0=0.04109, ω1=1.00000 | N/A |
| M2a | 35 | -6186.240980 | p0=0.93161, p1=0.06839, p2=0.00000, ω0=0.04109, ω1=1.00000, ω2=7.36484 | 77 M 0.528 |
| M7 | 33 | -6110.449895 | p=0.24894, q=3.20526 | N/A |
| M8 | 35 | -6110.450110 | p0=0.99999, p=0.24895, q=3.20578, p1=0.00001, ω=1.00000 | 77 M 0.604 |
| Branch | 33 | -6253.397862 | ω0=0.05743, ω1=1.17665 | N/A |
| Branch null | 32 | -6253.397861 | ω0=0.05743, ω1= 1.00000 | N/A |
| Branch-site | 35 | -6168.806096 | site class 0 1 2a 2b  proportion 0.87218 0.06479 0.05867 0.00436  background ω 0.03915 1.00000 0.03915 1.00000  foreground ω 0.03915 1.00000 16.34105 16.34105 | 102 C 0.889  120 S 0.560  123 S 0.987*  141 C 0.934  165 M 0.934  175 I 0.639  185 C 0.996**  219 M 0.932  221 S 0.572  305 Q 0.989*  306 G 0.758  308 A 0.950*  313 N 0.942  348 T 0.646 |
| Branch-site null | 34 | -6173.515909 | site class 0 1 2a 2b  proportion 0.85485 0.06432 0.07517 0.00566  background ω 0.03785 1.00000 0.03785 1.00000  foreground ω 0.03785 1.00000 1.00000 1.00000 | N/A |
| LW2 opsin gene |  |  |  |  |
| One-ratio (LW2) | 32 | -6135.444910 | ω=0.06902 | None |
| One-ratio (LW2=LW1) | 31 | -6138.400082 | The same as LW1 | N/A |
| M1a | 33 | -6063.923759 | p0= 0.92855, p1=0.07145, ω0=0.04966, ω1=1.00000 | N/A |
| M2a | 35 | -6063.923759 | p0=0.92855, p1=0.05433 p2=0.01712, ω0=0.04966, ω1=1.00000, ω2=1.00000 | 89 A 0.687 |
| M7 | 33 | -6024.654141 | p=0.37881, q=4.00534 | N/A |
| M8 | 35 | -6021.812481 | p0= 0.98371, p=0.44489, q=5.60448, p1=0.01629, ω=1.00000 | 89 A 0.906  195 I 0.626  198 L 0.594 |
| Branch | 33 | -6135.444963 | ω0=0.06902, ω0=1.82775 | N/A |
| Branch null | 32 | -6135.444955 | ω0=0.06902, ω0=1.00000 | N/A |
| Branch-site | 35 | -6045.583832 | site class 0 1 2a 2b  proportion 0.87432 0.06503 0.05644 0.00420  background ω 0.04825 1.00000 0.04825 1.00000  foreground ω 0.04825 1.00000 141.81634 141.81634 | 103 N 0.966*  135 W 0.909  169 Y 0.965*  187 T 0.522  199 H 0.923  234 G 0.995**  259 H 0.904  288 Q 0.960*  289 S 0.996**  327 Q 0.502  346 C 0.995** |
| Branch-site null | 34 | -6052.379094 | site class 0 1 2a 2b  proportion 0.86756 0.06570 0.06204 0.00470  background ω 0.04630 1.00000 0.04630 1.00000  foreground ω 0.04630 1.00000 1.00000 1.00000 | N/A |
| Blue opsin gene |  |  |  |  |
| One-ratio (Blue) | 32 | -6292.648374 | ω=0.05024 | None |
| M1a | 33 | -6271.413423 | p0=0.98061, p1=0.01939, ω0=0.04484, ω1=1.00000 | N/A |
| M2a | 35 | -6271.413423 | P0=0.98061, p1=0.01939, p2=0.00000, ω0=0.04484, ω1=1.00000, ω2= 43.1348 | None |
| M7 | 33 | -6224.195469 | p= 0.43783, q=7.17466 | N/A |
| M8 | 35 | -6223.828213 | p0= 0.99533, p=0.46348, q=8.01476, p1=0.00467, ω=1.00000 | None |
| Branch | 33 | -6292.648477 | ω0=0.05024, ω1=2.05602 | N/A |
| Branch null | 32 | -6292.648459 | ω0=0.05024, ω1=1.00000 | N/A |
| Branch-site | 35 | -6266.857242 | site class 0 1 2a 2b  proportion 0.94746 0.01937 0.03251 0.00066  background ω 0.04386 1.00000 0.04386 1.00000  foreground ω 0.04386 1.00000 72.53886 72.53886 | 43 H 0.808  146 S 0.881  152 S 0.898  231 A 0.536  239 C 0.721  302 C 0.839 |
| Branch-site null | 34 | -6268.148685 | site class 0 1 2a 2b  proportion 0.93875 0.01909 0.04131 0.00084  background ω 0.04323 1.00000 0.04323 1.00000  foreground ω 0.04323 1.00000 1.00000 1.00000 | N/A |
| UV opsin gene |  |  |  |  |
| One-ratio (UV) | 32 | -6314.695899 | ω=0.04998 | None |
| M1a | 33 | -6266.454843 | p0=0.94353, p1=0.05647, ω0=0.03544, ω1=1.00000 | N//A |
| M2a | 35 | -6266.454843 | p0=0.94353, p1=0.03521, p2=0.02126, ω0=0.03544, ω1=1.00000, ω2=1.00000 | 229 I 0.727 |
| M7 | 33 | -6197.763585 | p=0.23406, q= 3.62738 | N/A |
| M8 | 35 | -6194.448793 | p0= 0.99705, p=0.24663, q=4.16413, p1=0.00295, ω= 2.20391 | 229 I 0.925 |
| Branch | 33 | -6297.803852 | ω0=0.05716, ω1=0.00160 | N/A |
| Branch null | 32 | -6314.696196 | ω0=0.04998, ω1=1.00000 | N/A |
| Branch-site | 35 | -6266.454843 | site class 0 1 2a 2b  proportion 0.94353 0.05647 0.00000 0.00000  background ω 0.03544 1.00000 0.03544 1.00000  foreground ω 0.03544 1.00000 1.00000 1.00000 | None |
| Branch-site null | 34 | -6266.454843 | site class 0 1 2a 2b  proportion 0.94343 0.05646 0.00010 0.00001  background ω 0.03544 1.00000 0.03544 1.00000  foreground ω 0.03544 1.00000 1.00000 1.00000 | N/A |
