# Supplementary material for: Evolution and Expression Plasticity of Opsin Genes in a Fig Pollinator, Ceratosolen solmsi
Source: PLoS One. 2013 Jan 16;8(1):e53907. doi: 10.1371/journal.pone.0053907 (PMC3547053; doi:10.1371/journal.pone.0053907)
Supplement: Table S5 — Likelihood ratio tests (LRTs) for positive selection of fig pollinator opsin genes. (DOC) [file pone.0053907.s011.doc]

**Table S5 Likelihood ratio tests (LRTs) for positive selection of fig pollinator opsin genes.**

| Comparisons | 2ΔLnL | df | *P-value* |
| --- | --- | --- | --- |
| LW1 opsin gene |  |  |  |
| M2a vs M1a | 0 | 2 | 1 |
| M8 vs M7 | 0.00043 | 2 | 0.999785 |
| Branch vs Branch null | 0.000002 | 1 | 0.998872 |
| Branch-site vs Branch-site null | 9.419626 | 1 | **<0.001** |
| LW2 opsin gene |  |  |  |
| M0 (LW2) vs M0 (LW2=LW1) | 5.910344 | 1 | ***P=*0.015** |
| M2a vs M1a | 0 | 2 | 1 |
| M8 vs M7 | 5.68332 | 2 | **<0.05** |
| Branch vs Branch null | 0.000016 | 1 | 0.996808 |
| Branch-site vs Branch-site null | 13.590524 | 1 | **<0.001** |
| Blue opsin gene |  |  |  |
| M2a vs M1a | 0 | 2 | 1 |
| M8 vs M7 | 0.734512 | 2 | 0.346316 |
| Branch vs Branch null | 0.000036 | 1 | 0.995213 |
| Branch-site vs Branch-site null | 2.582886 | 1 | 0.0540125 |
| UV opsin gene |  |  |  |
| M2a vs M1a | 0 | 2 | 1 |
| M8 vs M7 | 6.629584 | 2 | 0.018171 |
| Branch vs Branch null | 33.784688 | 1 | **<0.001** |
| Branch-site vs Branch-site null | 0 | 1 | 0.5 |
