# Supplementary material for: Evolution and Expression Plasticity of Opsin Genes in a Fig Pollinator, Ceratosolen solmsi
Source: PLoS One. 2013 Jan 16;8(1):e53907. doi: 10.1371/journal.pone.0053907 (PMC3547053; doi:10.1371/journal.pone.0053907)
Supplement: Table S6 — Descriptions of primer pairs used for RT-qPCR analysis. (DOC) [file pone.0053907.s012.doc]

**Table S6 Descriptions of primer pairs used for RT-qPCR analysis.**

| Gene | Forward primer (5’-3’) | Reverse primer (5’-3’) | Amplicon length (bp) | Es(%) | R2 |
| --- | --- | --- | --- | --- | --- |
| RPL13a | CTGCTCGTGGTCCTTTCCATTTTC | GCATCCTTGCCTCTTTGTGTCTTG | 97 | 104.4 | 0.997 |
| UBC | GAAGCGGATCAACAAGGAACT | GGACTGTCAGGTGGACCCATAAT | 123 | 96.6 | 0.992 |
| LW1 opsin | CAGAGAACAAACTCGCCAAGGT | ACAGTGGAGACGGCATCGG | 268 | 102.5 | 0.995 |
| LW2 opsin | GGGCTTTGGGTCCTCTAATGTGT | ACAAGATGTCATGTTGCCTTCGG | 254 | 94.0 | 0.999 |
| Blue opsin | ACTAATGCGGCTATTGCCTACG | AATCCCTCTGTGGCATATCGG | 170 | 105.7 | 0.997 |
| UV opsin | CTACCTCACCGACTCCAGCGA | GCCTTGGCGATGCGGAC | 225 | 91.2 | 1.000 |
